# Ciliated epithelial cell differentiation at air–liquid interface and respiratory syncytial virus infection using animal-free media and substrates

**DOI:** 10.1183/23120541.00028-2025

**Published:** 2025-12-08

**Authors:** Machaela Palor, Elizabeth K. Haughey, Jenny Herbert, Christopher O'Callaghan, Rosalind L. Smyth, Paolo De Coppi, Robert E. Hynds, Claire M. Smith

**Affiliations:** 1 Infection, Immunity and Inflammation, UCL Great Ormond Street Institute of Child Health, University College London, London, UK; 2 School of Medical Sciences, Faculty of Biology, Medicine and Health, University of Manchester, Manchester, UK; 3 Epithelial Cell Biology in ENT Research (EpiCENTR) Group, Great Ormond Street UCL Institute of Child Health, University College London, London, UK

## Abstract

**Background:**

Animal-derived components in cell culture, such as fetal bovine serum and extracellular matrix proteins, pose ethical concerns and contribute to variability in experimental outcomes. This study explores the use of animal-free cell culture media and substrates to support the growth and differentiation of primary human bronchial epithelial cells, as well as their infection by respiratory syncytial virus (RSV).

**Methods:**

We evaluated the performance of jellyfish collagen 0 and recombinant extracellular matrix proteins as alternatives to traditional mammalian substrates. Additionally, we assessed the use of animal-free medium and human serum (HS) in viral propagation using HEp2 cells.

**Results:**

The use of animal-free medium, matrix proteins and HS can support primary epithelial cell growth and differentiation with high levels of ciliation and barrier integrity. RSV propagation in animal-free medium produced an increase in viral titres, indicating the potential of these systems for antiviral research.

**Conclusion:**

Transitioning to include more animal-free medium and substrates for primary cell culture and viral propagation will help improve the ethical standing of research and offer more human-relevant models for studying viral diseases in the future.

## Shareable abstract

**Animal-free cell culture supports epithelial cell growth, differentiation and RSV infection, offering an ethical, reliable and human-relevant model for viral research** https://bit.ly/4lbJI4L

## Introduction

Cell culture systems are essential for studying cellular processes and viral infections. Traditionally, these systems use animal-derived products such as fetal bovine serum (FBS) and collagen to provide nutrients and support for cell growth. However, these components raise ethical concerns and introduce variability, as well as potential contamination risks [1]. The need for animal-free alternatives is evident, aiming to reduce reliance on animal products while enhancing experimental reproducibility​.

In December 2022, the US enacted the Food and Drug Administration Modernization Act 2.0, allowing alternatives to animal testing (such as cell-based assays, organoids and computational models) to play a more prominent role in the drug approval process. This change in legislation is accelerating the transition to animal-free research methodologies, including those that do not involve the use of animals or animal-derived products [2, 3]. In cell culture, animal-free conditions (those completely devoid of animal-derived components) are thought to be important for enhancing assay reproducibility and regulatory compliance, especially in the context of producing cell-based therapies [4, 5].

Animal-free culture systems aim to replace traditional reagents, such as FBS and animal-derived extracellular matrix (ECM) proteins, with alternatives such as human serum (HS) and human-derived ECM proteins. While these alternatives offer improved compatibility and reduced immunogenicity compared with their animal counterparts, they are constrained by factors such as availability, variability and high costs. Consequently, options such as defined serum-free formulations and recombinant ECM proteins, such as iMatrix-511, are being explored to effectively support cell growth and differentiation [1, 6].

Respiratory syncytial virus (RSV) is a leading cause of acute lower respiratory tract infections, particularly in infants, young children and the elderly, representing a significant global health burden [7]. RSV research and the development of antiviral therapies or vaccines often relies on animal models, including mice and cotton rats, to study antiviral therapies and host–pathogen interactions. However, these do not accurately replicate the virus or reproduce the pathology of disease observed in humans. This lack of translational relevance has contributed to limited major breakthroughs in understanding RSV infection mechanisms or developing effective therapeutic strategies [8, 9].

To address these limitations, more complex *in vitro* models are needed to study viral infection mechanisms and immune involvement. Inverted air–liquid interface (ALI) cultures provide an alternative approach by enabling interactions between epithelial cells and immune cells in a physiologically relevant orientation [10, 11]. Despite their advantages, these models present challenges related to cell adhesion and differentiation compared with traditional gravity-assisted conditions, which can affect barrier formation and overall model stability.

In this study, our aim was to demonstrate the feasibility of animal-free cell culture media and substrates for supporting the growth and differentiation of primary paediatric human bronchial epithelial cells (BECs) using the inverted ALI system and for propagating RSV for infection studies. Establishing feasibility in these complex models could pave the way for easier adaptation to simpler traditional models, promoting broader adoption of animal-free methods in airway research.

## Methods

### Animal-free cell attachment and growth

Primary paediatric (2-year-old, white female) BECs were purchased from Epithelix Sàrl (Geneva, Switzerland). While the exact conditions of their initial culture by the supplier are unknown, it is likely that they were not animal-free. BECs were grown on human placenta collagen IV (Merck, Germany) or mammalian-free substrates, specifically jellyfish collagen 0 (Jellagen, Cardiff, UK) or iMatrix-511, a recombinant extracellular matrix protein (AMSBIO, Oxford, UK), or rat tail collagen I (Corning, NY, USA), which is commonly used for cell culture applications. The number of viable cells was determined at 24, 48, 72 and 96 h by detaching the cells using TrypLE Express (Thermo Fisher Scientific, UK) and counting viable cells using the trypan blue exclusion method. Cell proliferation was also monitored using the zenCELL owl incubator microscope (LabLogic, UK), as shown in supplementary figure S1. While metabolic assays such as MTT or resazurin could provide additional insights, trypan blue is a validated method for assessing BEC viability [12, 13].

BECs were also grown in two types of medium: PromoCell Airway Epithelial Cell (AEC) Growth Medium (AECGM) (PromoCell, Heidelberg, Germany), which is based on our previously published model [11] or Epithelix Human Airway Epithelial Cell/Human Small Airway Epithelial Cell (hAEC/hSAEC) xeno-free culture medium (hAECM/hSAECM; Epithelix Sàrl, Geneva, Switzerland).

### Cell propagation and ALI differentiation

Primary paediatric BECs were co-cultured on mitotically inactivated 3T3-J2 feeder cells [14] in primary AEC culture medium (3T3-Y conditions) composed of 325 mL DMEM, 125 mL Ham's F-12 (Thermo Fisher Scientific, Dartford, UK), 9% HS (Merck, Darmstadt, Germany), 1% penicillin–streptomycin, 10 µg·mL^−1^ gentamicin (Thermo Fisher Scientific, UK), 25 ng·mL^−1^ hydrocortisone (Merck, Germany), 0.125 ng·mL^−1^ recombinant human epidermal growth factor (Thermo Fisher Scientific, UK), 5 µg·mL^−1^ bovine insulin (I5500, Merck, Darmstadt, Germany), 5 µM Y-27632 Rho-associated coiled-coil kinase (ROCK) inhibitor (Enzo Life Sciences, USA), 250 ng·mL^−1^ amphotericin B (Thermo Fisher Scientific, UK) and 0.1 nM cholera toxin (Merck, Germany).

BECs were then cultured at the ALI in complete PneumaCult^TM^-ALI medium (STEMCELL Technologies, Vancouver, Canada) to promote differentiation, a process that was monitored over a period of 28 days.

### Evaluation of barrier integrity

At 28-days post-ALI, the integrity of the epithelial barrier was evaluated using trans-epithelial electrical resistance (TEER) measurements and dextran permeability assays. TEER was measured with an Epithelial Volt/Ohm Meter 3 (EVOM3, World Precision Instruments), adjusting for the membrane area. For dextran permeability, ALI cultures were placed in Hanks’ balanced salt solution containing calcium and magnesium (HBSS+/+) plus Texas Red™-dextran (Thermo Fisher Scientific, UK) at 100 µg·mL^−1^ for 20 min in the dark. Then, 100 µL of standards and supernatant were transferred to a solid black 96-well plate in triplicate and fluorescence was measured with a microplate reader at an excitation/emission wavelength of 595/615 nm. A membrane without cells served as a control. The translocated dextran concentration, derived from a standard curve, indicated paracellular permeability.

### Ciliary beat frequency

Fast timelapse videos were acquired using a Nikon Eclipse Ti-E inverted microscope (Nikon, Japan) equipped with an ORCA-Flash 4.0 digital CMOS camera (Hamamatsu, Japan) and processed using Fiji image analysis software [15]. Ciliary beat frequency (CBF) was then measured with an automated ciliR code for fast Fourier transform of the data (using R), as previously described [16].

### Flow cytometric analysis

For flow cytometry, ALI cultures were rinsed apically with Dulbecco's PBS (DPBS; Thermo Fisher Scientific, UK) and cells were dissociated from the membrane using TrypLE Express (Thermo Fisher Scientific, UK). Cells were blocked in Human TruStain FcX™ (BioLegend, USA) diluted 1:50 in DPBS for 10 min at 4°C to minimise Fc receptor nonspecific binding and stained with LIVE/DEAD™ fixable near IR dye to determine the viability of cells (Thermo Fisher Scientific, UK).

Cells were washed by adding 100 μL FACS buffer (1× DPBS, 1% HS and 1 mM EDTA) and centrifuging at 300×g for 5 min, then stained with surface markers CD49f-PE/Dazzle™-594 (BioLegend, San Diego, CA, USA), CD271-BV421 (BD Biosciences, Franklin Lakes, NJ, USA), TSPAN8-BV711 (BD Biosciences, Franklin Lakes, NJ, USA) and CD66c-PE (BD Biosciences, USA) in 50 μL FACS buffer for 20 min at 4°C in the dark (table 1). Cells were washed by adding 100 μL FACS buffer and centrifuging at 300×g for 5 min, fixed with 4% (v/v) paraformaldehyde (PFA; Thermo Fisher Scientific, UK) for 20 min at 4°C and then permeabilised by washing in permeabilisation buffer (Thermo Fisher Scientific, UK). For intracellular staining, cells were resuspended in 50 μL permeabilisation buffer containing MUC5AC-FITC (Novus Biologicals, USA) and acetyl-α-tubulin-Alexa Fluor™ 647 (Cell Signaling Technology, Danvers, MA, USA) for 20 min at 4°C in the dark (table 1). After a final wash in permeabilisation buffer, cells were resuspended in 200 μL FACS buffer and analysed using a CytoFLEX S flow cytometer.

**TABLE 1:** Antibodies used for flow cytometric analysis of differentiated bronchial epithelial cells

Antibody	Target	Fluorophore	Supplier	Host	Animal-derived	Dilution
**CD49f (ITGA6)**	Basal	PE-Dazzle™ -594	BioLegend (313626)	Rat	Yes^#^	1:50
**CD271 (NGFR)**	Basal	BV421	BD Biosciences (562562)	Mouse	Yes^#^	1:20
**TSPAN8**	Goblet	BV711	BD Biosciences (748227)	Rat	Yes^#^	1:100
**CD66c**	Secretory	PE	BD Biosciences (551478)	Mouse	Yes^#^	1:100
**MUC5AC**	Goblet	FITC	Novus Biologicals (NBP2-32732AF488)	Mouse	Yes^#^	1:50
**Acetyl-α-tubulin**	Ciliated	Alexa Fluor™ 647	Cell Signaling Technology (81502)	Rabbit	Yes^#^	1:50

^#^: To allow direct comparisons with previously published work.

Basal cells were defined as the proportion of CD49f/CD271 double-positive cells in the live cell population. Ciliated cells were then gated from the nonbasal cell population based on acetyl-α-tubulin^hi^ and CD66c^lo^ expression, whereas goblet cells were further separated from the nonciliated population based on positive expression for MUC5AC [17].

### Fluorescence confocal imaging

ALI cultures were fixed with 4% (v/v) PFA in DPBS for 30 min at room temperature, washed twice in DPBS and blocked and permeabilised in 3× Animal-Free Blocker® (Vector Laboratories, Newark, CA, USA) with 0.1% Triton™ X-100 (Merck, Germany) for 1 h at room temperature. Membranes were then carefully removed using a scalpel and incubated with fluorescently labelled recombinant antibodies diluted in 1× Animal-Free Blocker® with 0.1% Triton™ X-100 for 1 h at room temperature (table 2). For ciliated cells, membranes were treated with fluorescently labelled wheat germ agglutinin, an animal-free carbohydrate-binding lectin, which has previously been used in studies involving ciliated cells of the respiratory epithelium [18, 19]. After three washes in DPBS, membranes were incubated with 4′,6-diamidino-2-phenylindole (DAPI) for 10 min at room temperature then mounted onto a glass slide with propyl gallate mounting medium (Merck, Germany).

**TABLE 2:** Antibodies or conjugate used for immunofluorescence staining

Antibody/conjugate	Target	Fluorophore	Supplier	Animal-derived	Dilution
**Keratin 5 (KRT5)**	Basal cells	APC	Miltenyi Biotec (130-127-016)	No	1:50
**MUC5AC**	Goblet cells	PE	Miltenyi Biotec (130-127-552)	No	1:50
**Wheat germ agglutinin**	Ciliated cells	Fluorescein	2BScientific (FL-1021-5)	No	1:100

Slides were imaged using a ×20 Plan Achromat Objective and a Zeiss LSM710 confocal microscope with the pinhole set at 1 Airy unit.

### Viral propagation

Recombinant green fluorescent protein (GFP)-tagged RSV A2 strain was kindly provided by Professor Jean-François Eléouët (Unité de Virologie Immunologie Moléculaires, France) [20]. RSV propagation was evaluated using Human Epithelial Type 2 (HEp-2, CCL-23™) cells from ATCC (Virginia, USA). These were maintained in VP-SFM (Thermo Fisher Scientific, UK) supplemented with 4 mM l-glutamine (Thermo Fisher Scientific, UK) and 0.5% penicillin–streptomycin for virus production or DMEM with 2% HS and 0.5% penicillin–streptomycin for viral quantification. The efficiency of RSV infection and replication was assessed by quantifying viral titres at different time points post-infection. This approach was designed to test the efficacy of serum-free medium in supporting viral growth and to compare it with traditional serum-containing medium.

### BD cytometric bead array analysis

This assay was carried out using a cytometric bead array (CBA) (BD Biosciences, USA); antibody-coated beads of known size and fluorescence are used to detect multiple proteins in each sample. This assay was carried out as described in the manufacturer's protocol to quantify human cytokines such as human interleukin (IL)-6 (A7, 558276, BD Biosciences), human IL-8 (A9, 558277, BD Biosciences) and human IP-10 (B5, 558280, BD Biosciences), in supernatant obtained from mock- or RSV-infected cultures. Test samples were diluted 1:10 in assay diluent to ensure their median fluorescence values fall within the range of the generated standard curve. Then, 50 μL of each standard and test sample were incubated with 50 μL mixed capture beads for 1 h at room temperature on an orbital shaker. 50 μL mixed PE detection reagent was then added to each assay tube for a further 2 h. Beads were washed by adding 50 μL wash buffer (1×DPBS, 1% HS and 0.09% sodium azide) and centrifuging at 200×g for 5 min. Beads were resuspended in 200 μL wash buffer and analysed using a BD FACSymphony A5 flow cytometer.

### Real-time quantitative PCR for RSV detection

Viral RNA was extracted from supernatant using the QIAamp Viral RNA Mini kit (QIAGEN, Germany) as per the manufacturer's instructions and quantified with a Thermo Scientific Nanodrop 1000 (Thermo Fisher Scientific, UK). 100 ng total RNA was then used to synthesise cDNA using the High-Capacity RNA-to-cDNA kit (Thermo Fisher Scientific, UK) in a final reaction volume of 40 μL. Real-time quantitative PCR (qPCR) was performed using TaqMan Universal Master Mix II, with UNG (Thermo Fisher Scientific, UK) containing specific primers for RSV-A N protein and a fluorescently labelled probe (table 3) [21]. Primers and probes were used at final concentrations of 900 nM and 250 nM, respectively. Samples were run on an AB Biosystems StepOnePlus Real-Time PCR System (Thermo Fisher Scientific, UK); cDNA was denatured at 95°C for 10 min and amplified by 40 cycles (95°C for 15 s and 60°C for 1 min). The viral load was extrapolated from a standard curve generated by performing ten-fold serial dilutions of a plasmid containing the N protein sequence starting at 1×10^6^ copies to one copy [22].

**TABLE 3:** Primers and probes used for respiratory syncytial virus (RSV) real-time quantitative PCR

Primer/probe	Sequence 5′–3′
**RSV-A forward primer**	CTCAATTTCCTCACTTCTCCAGTGT
**RSV-A reverse primer**	CTTGATTCCTCGGTGTACCTCTGT
**RSV-A probe**	(6FAM) TCCCATTATGCCTAGGCCAGCAGCA (TAMRA)

### Data analysis

Data from cell viability assays, TEER measurements and viral titres were statistically analysed using ANOVA and mixed-effects models to compare the performance of animal-free systems with traditional methods. The primary focus was on cell viability, differentiation efficiency, TEER values and RSV propagation efficiency. Flow cytometry data were analysed using FlowJo v.10 (Tree Star, San Carlos, CA, USA).

## Results

### Jellyfish collagen 0 best supports human BEC attachment

To determine alternative ECM substrates for the expansion of human AECs, primary paediatric BECs were first cultured on different ECM proteins in PromoCell AECGM. These included the standard rat tail collagen I, commonly used for ALI culture, human placenta collagen IV and two alternatives of nonmammalian ECM: jellyfish collagen 0 and iMatrix-511, which is recombinantly expressed in an epithelial cell line.

**FIGURE 1:**
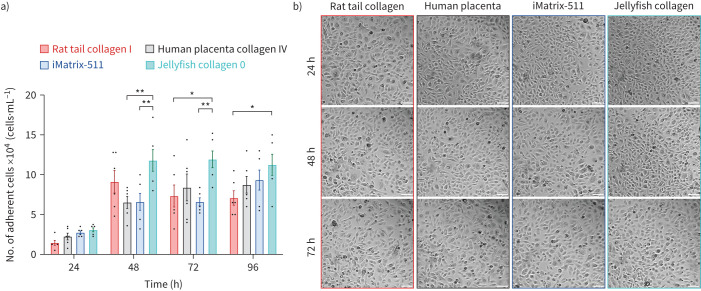
Comparing animal-free and conventional (rat tail collagen) substrates for bronchial epithelial cell (BEC) attachment. BECs were grown on different surfaces: rat tail collagen I, human placenta collagen IV, jellyfish collagen 0 and iMatrix-511. a) Number of adherent BECs at 24, 48, 72 and 96 h. Error bars represent mean±sem of n=6 independent experiments. b) Representative timelapse phase-contrast images at 24–72 h post-seeding. Scale bars, 100 μm. *: p<0.05, **: p<0.01 (two-way ANOVA with Tukey *post hoc* test).

The adhesion of BECs at 24, 48, 72 and 96 h post-seeding was assessed by detaching adherent cells and counting viable cells using the trypan blue exclusion method (figure 1a). We recovered more viable BECs from plates coated in jellyfish collagen 0 at 48, 72 and 96 h (mean±sem of 118 000±13 653, 119 333±10 503 and 112 501±13 276 cells·mL^−1^) in comparison with plates coated in rat tail collagen I (73 333±13 844 and 70 833±9075 cells·mL^−1^), human placenta collagen IV (65 333±7566 cells·mL^−1^) and iMatrix-511 (66 000±10 570 and 66 000±5033 cells·mL^−1^) (p<0.05, n=6 independent experiments). We found no difference in the number of recovered BECs grown on rat tail collagen I compared with either human placenta collagen IV or iMatrix-511.

BECs were also assessed using brightfield microscopy, with no noticeable changes in cell morphology detected between the cell attachment substrates (figure 1b).

In summary, human BECs can attach to plates coated in ECM-derived from human and nonmammalian sources with no negative impact on cell viability or morphology. As BECs adhered in greater numbers to jellyfish collagen 0 compared with the standard rat tail collagen I and other cell attachment substrates tested, this substrate was used for subsequent comparison studies.

### Animal-free medium supports BEC culture but less effectively than a 3T3+Y feeder cell-based method

**FIGURE 2:**
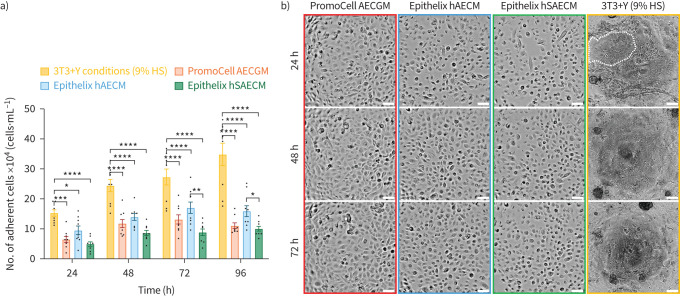
Primary paediatric bronchial epithelial cells (BECs) can be cultured in human serum (HS) or animal-free medium. BECs were either grown in co-culture with feeder cells in 3T3+Y with 9% HS conditions (gold bars) or on jellyfish collagen 0 using two types of medium: PromoCell Airway Epithelial Cell (AEC) Growth Medium (AECGM) (red bars) or Epithelix Human Airway Epithelial Cell/Human Small Airway Epithelial Cell (hAECM/hSAECM) (blue/green bars). a) Number of adherent BECs at 24, 48, 72 and 96 h. Error bars represent mean±sem of n=9 independent experiments. b) Representative timelapse phase-contrast images at 24–72 h. BECs exhibited a cobblestone-like morphology (dotted line) in co-culture with 3T3-J2 feeder cells. Scale bars, 100 μm.*: p<0.05, **: p<0.01, ***: p<0.001, ****: p<0.0001 (two-way ANOVA with Tukey *post hoc* test).

The growth of primary paediatric BECs in different animal-free culture medium, including PromoCell AECGM, Epithelix hAECM, hSAECM and the 3T3+Y method supplemented with HS was then investigated. Cell viability was quantified at 24, 48, 72 and 96 h using the trypan blue exclusion method. The findings indicate significantly higher numbers of viable BECs across all time points when cultured using the 3T3+Y method supplemented with HS (152 889±10 536, 244 444±20 314, 272 889±26 548 and 348 444±37 217 cells·mL^−1^) compared with the other growth media tested (p<0.05, n=9 independent experiments; figure 2a) [14, 23]. Additionally, the number of viable BECs in Epithelix hAECM were also significantly greater at 72 and 96 h (170 222±19 103 and 159 111±18 255 cells·mL^−1^) compared with hSAECM (88 444±10 943 and 100 444±7985 cells·mL^−1^) (p<0.05, n=9 independent experiments). No significant differences were observed in growth between BECs maintained in PromoCell AECGM and those in either Epithelix hAECM or hSAECM. This was confirmed by capturing real-time phase-contrast images and using automated image processing algorithms (supplementary figure S1).

Cell morphology was also assessed using brightfield microscopy; BECs grown on a feeder layer in 3T3+Y conditions exhibited a characteristic cobblestone-like appearance with small epithelial colonies, while BECs cultured on jellyfish collagen 0 in either PromoCell AECGM, Epithelix hAECM or hSAECM were larger and did not form compact cell clusters (figure 2b).

In summary, we found that human BECs can be cultured with comparable cell numbers using different animal-free media, but the number of viable cells was approximately half that achieved with the 3T3+Y method. Given the importance of attaining optimal phenotypic and functional end-points, such as differentiation, CBF and TEER, BECs cultured under 3T3+Y conditions supplemented with HS are recommended for further cell propagation.

### Animal-free matrix proteins support epithelial cell differentiation at ALI, with optimal ciliation observed on human placenta collagen IV

To evaluate whether the use of animal-free components affects the capacity of human BECs to differentiate into ciliated epithelial cells (the target of RSV infection [24]) BECs grown in 3T3+Y conditions or cultured on jellyfish collagen 0 in Epithelix hAECM were seeded on membrane inserts coated with either rat tail collagen I, human placenta collagen IV and jellyfish collagen 0. We found that BECs that had been cultured on jellyfish collagen 0 in Epithelix hAECM did not adhere to the collagen-coated membrane inserts with the cells detaching upon removal of the apical medium during the air-lifting process (data not shown). BECs grown in 3T3+Y conditions survived up to 4 weeks at the ALI, but exhibited either no ciliation on jellyfish collagen 0, low-level ciliation (<5 areas with motile cilia) on rat tail collagen I or high-level ciliation (>5 areas with motile cilia) on human placenta collagen IV as based on previously established morphological criteria and observed under phase-contrast microscopy [25] (table 4). A representative slow-motion video of cells grown on human placenta collagen IV is shown in Video 1.

**TABLE 4:** Differentiation of inverted air–liquid interface cultures

Cell attachment substrate	Level of ciliation
**Rat tail collagen I**	+
**Human placenta collagen IV**	++
**Jellyfish collagen 0**	−

+: <5 areas with motile cilia observed; ++: >5 areas with motile cilia observed; −: no ciliation observed.

**FIGURE 3:**
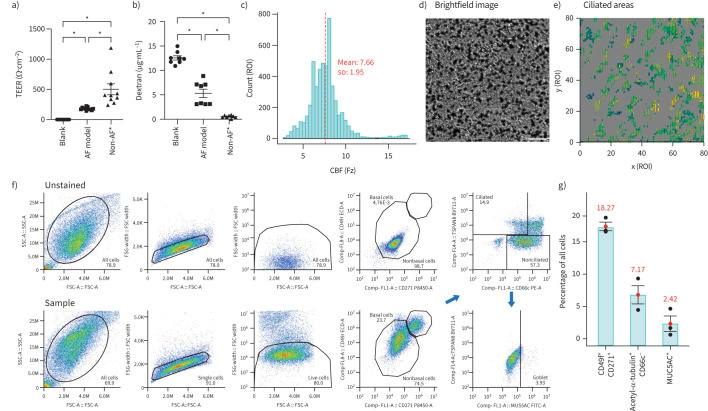
Barrier integrity and ciliated epithelial cell differentiation of human bronchial epithelial cells (BECs) at air–liquid interface (ALI). a,b) Epithelial integrity of BECs differentiated from one donor at ALI on membrane inserts coated with human placenta collagen IV for 4 weeks (n=8) compared with our non-animal-free (AF)model (n=10) [11] that contains an additional Matrigel (mouse-derived) layer. Barrier integrity was measured by trans-epithelial electrical resistance (TEER) (Ω·cm^−2^) (a) and dextran permeability (μg·mL^−1^) (b). Error bars represent mean±sem of n=8–10 independent Transwells. *: p<0.05 (unpaired ANOVA with Benjamini–Hochberg correction for multiple comparisons). c) Histogram of ciliary beat frequency (CBF) (Hz) was quantified *via* high-speed video microscopy of 6400 regions of interest (ROIs) from five independent areas (one airway epithelial cell (AEC) donor). The red dotted line represents the mean CBF value of all areas. d,e) Representative brightfield image (d) and activity map (e) of one area (field of view) to show the position and distribution of motile ciliated cells. Scale bar, 50 μm. f) Example flow cytometry gating strategy to determine the population of basal, ciliated and goblet cells in inverted ALI cultures. Basal cells were defined as the proportion of CD49f/CD127 double-positive cells in the live cell population. Ciliated cells were then gated from the nonbasal cell population based on acetyl-α-tubulin^+^ and CD66c^−^ expression, whereas goblet cells were further separated from the nonciliated population based on positive expression for MUC5AC. g) Proportion (%) of basal (CD49f^+^CD271^+^), ciliated (acetyl-α-tubulin^+^ CD66c^−^) and goblet (MUC5AC^+^) cells from one AEC donor (n=3 wells). Red dot and error bars represent mean±sem of n=3 wells.

However, human BECs differentiated on human placenta collagen IV-coated membrane inserts remained adhered and, after 4 weeks, exhibited a mean TEER value of 180.7±6.7 Ω·cm^−2^ (figure 3a). Although this was significantly lower than the 348.2±61.8 Ω·cm^−2^ reported in our previous animal-component-based model, which included an additional Matrigel (mouse-derived) layer [11], it was significantly higher than the blank, which indicates that the animal-free model still establishes a restrictive epithelial barrier. This was further supported by a reduced permeability to 3 kDa Texas red-dextran (5.3±0.8 μg·mL^−1^) compared with empty membrane inserts (12.6±0.5 μg·mL^−1^) (p<0.0001, n=8 independent experiments; figure 3b). Beating cilia were observed (Video 1), indicating cell differentiation and polarisation and CBF was quantified *via* high-speed video microscopy, revealing that approximately 20% of ROIs had active cilia and a mean CBF of 7.66±1.95 Hz (figure 3c–e).

Flow cytometry analysis quantified the proportions of basal, ciliated and goblet cells in the ALI cultures, as described previously [17]. The cell gating technique is show in figure 3f. The proportions of each cell type were calculated from the total number of cells acquired per well, with basal cells (CD49f^+^CD271^+^) making up approximately 18% (4001±115 cells), ciliated cells (acetyl-α-tubulin^hi^CD66c^lo^) around 7% (1557±488 cells) and goblet cells (MUC5AC^+^) about 2% (549±519 cells) of the total cell population (21 951±1400 cells) (figure 3g). Other distinct populations were identified, including acetyl-α-tubulin^+^CD66c^+^ nonbasal cells and MUC5AC^−^ nonciliated cells, which are likely to represent secretory and intermediate progenitor populations for ciliated and goblet cells as revealed by single-cell RNA sequencing of human AECs during *in vitro* ALI differentiation [26]. The functional identity and homogeneity of these populations remain unclear and, thus, they were excluded from further analysis. ALI cultures were also stained for expression of cell-type markers using animal-free reagents. However, the resulting staining was weaker than expected and requires further optimisation (preliminary staining shown in supplementary figure S2).

In summary, human BECs were able to differentiate into multiple specialised cell types, including ciliated and goblet cells, when grown at the ALI on human placenta collagen IV-coated membranes (*i.e.* in animal-free conditions). This model enables the epithelial layer of the human airway to be the unit of study, allowing for the investigation of disease mechanisms during RSV infection, which preferentially target ciliated epithelial cells [27].

### VP-SFM supports RSV production in HEp-2 cells and successful infection of differentiated BECs in ALI cultures

**FIGURE 4:**
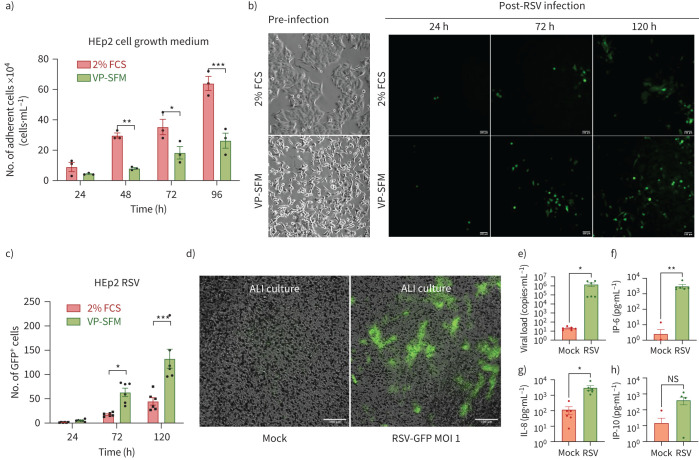
Respiratory syncytial virus (RSV) propagation and air–liquid interface (ALI) culture infection in animal-free conditions. a) Number of adherent Hep-2 cells at 24, 48, 72 and 96 h. Hep-2 cells were cultured in either DMEM supplemented with 2% fetal calf serum (FCS) or VP-SFM. b) Representative phase-contrast images of Hep2 pre-infection at 48 h post-seeding and representative fluorescent microscopy images to show green fluorescent protein (GFP) expression at 24, 72 and 120 h post-infection (multiplicity of infection (MOI) 0.01). c) The number of GFP-positive cells counted using ImageJ. Error bars represent mean±sem of n=3 donors and six technical repeats. *: p<0.05, **: p<0.01, ***: p<0.001 (two-way ANOVA with Bonferroni correction). Scale bars, 100 μm. d) Representative fluorescent microscopy images of mock- and RSV-infected bronchial epithelial cells (BECs) grown at ALI at 24 h post-infection (MOI 1). Scale bars, 100 μm. e) Real-time quantitative PCR analysis to determine RSV copy number in the apical supernatant of mock- and RSV-infected BECs at 24 h. Boxplots show viral load (copies·μL^−1^). The red dashed line represents the threshold for detecting the virus. Cytokine analysis of (f) interleukin (IL)-6, (g) IL-8 and (h) IP-10 in the apical supernatant of mock- and RSV-infected BECs at 24 h. and concentration (pg·mL^−1^) of n=6 independent experiments. *: p<0.05 (Wilcoxon signed-rank test); ns: not significant.

The next stage was to assess whether alternative animal-free media could support viral replication by the host cells and subsequent production of viral stocks for cell-infectivity assays and antiviral testing. Results showed that HEp-2 cells had a significantly higher viability in DMEM supplemented with 2% FBS at later time points (48 h, 296 670±16 670 cells·mL^−1^; 72 h, 353 330±50 440 cells·mL^−1^; and 96 h, 640 000±46 190 cells·mL^−1^) compared with VP-SFM (48 h, 80 000±5770 cells·mL^−1^; 72 h, 183 330±40 960 cells·mL^−1^, and 96 h, 263 330±49 780 cells·mL^−1^) (p<0.05, n=3 technical repeats; figure 4a). Additionally, HEp-2 cells cultured in VP-SFM underwent morphological changes, appearing more elongated and fibroblast-like compared with parental HEp-2 cells maintained in 2% FBS (figure 4b).

These cells were also tested for their susceptibility to infection with recombinant GFP-tagged RSV. Surprisingly, we found that despite their reduced growth in VP-SFM, HEp-2 cells showed a significantly higher number of infected, GFP-positive cells at 72 and 120 h (63±9 and 133±20 cells) compared with those maintained in 2% FBS (18±1 and 45±8 cells) (p<0.05, n=6 technical repeats; figure 4c).

Furthermore, we found that RSV propagated in HEp-2 cells cultured in VP-SFM could infect differentiated BECs grown at the ALI that were also grown under animal-free conditions. At 24 h post-infection GFP-positive cells were readily detected in RSV-infected BECs, while no GFP signal was observed in the mock-infected control (figure 4d). Apical supernatant from mock- and RSV-infected BECs were collected and analysed by real-time qPCR and flow cytometry to quantify viral RNA. As expected, a significantly higher viral load was detected in the apical supernatant of RSV-infected BECs (1.5×10^6^±6.8×10^5^ copies·μL^−1^), indicating the presence of virus, compared with mock-infected control cells (20.7±3.6 copies·μL^−1^) (figure 4e). RSV infection also led to a significantly higher concentration of IL-6 and IL-8 in the apical supernatant of RSV-infected BECs (3124±860 and 3119±1091 pg·mL^−1^) compared with mock-infected control cells (2.5±2.3 and 118±62.7 pg·mL^−1^, respectively) (figure 4f–h). No differences were observed in the levels of IP-10 in the apical supernatant of mock- or- RSV-infected BECs. Together, these findings support the use of VP-SFM as a viable animal-free alternative for culturing HEp-2 cells and producing RSV for research purposes.

## Discussion

The aim of this study was to replace the use of animal-derived media and substrates in the culture of a human-relevant *in vitro* model to study RSV infection. Different commercially available cell attachment substrates and culture media were investigated for growing human BECs and HEp2 cells.

Our findings highlight the potential use of jellyfish collagen 0 and human recombinant laminin-511 as alternatives to the traditionally used rat tail collagen I in the culture of primary BECs. These alternatives not only support comparable cell attachment but also address the ethical and safety concerns associated with mammalian collagen. Jellyfish collagen 0 has previously been used to successfully culture multiple cell types, including human primary cells and stem cells with comparable immunogenic responses and cytokine release profiles to mammalian collagen *in vitro* [28]. Human recombinant laminin-511, on the other hand, enables feeder-free culture and provides superior adhesion of human-induced pluripotent stem cells and human embryonic stem cells than full-length laminin or Matrigel™ [29].

While jellyfish collagen 0 showed promise as an alternative for growing human BECs in two-dimensional culture, it was unsuitable for differentiating BECs at the ALI, as cells detached from the membrane upon removal of the apical medium. This may be due to morphological differences between human BECs grown in monoculture, which are larger and less compact, compared with those co-cultured with feeder cells and treated with Y-27632 (a ROCK inhibitor), which are compacted by the fibroblast colonies as shown in figure 2b. These findings suggests that the culture conditions, including the quality of expanded basal BECs, influence cell survival and differentiation potential. Indeed, Y-27632 has been shown to enhance basal BEC proliferation *in vitro* without compromising subsequent ciliated epithelial differentiation [30, 31]. This effect is associated with cell rounding, reduced cell size and the localisation of E-cadherin and actin at cell–cell junctions upon confluence [30]. As the composition of most commercially available culture media is proprietary, it is difficult to conclude whether the differences observed here are due to any one factor or component in the medium. However, in our study, human BECs treated with Y-27632 in the presence of a 3T3-J2 feeder layer provided the most optimal conditions for achieving phenotypic and functional end-points. While the 3T3-J2 feeder cells were cultured in medium supplemented with HS, their initial derivation involved bovine serum [14]. The extent to which HS affects long-term feeder cell function warrants further investigation.

Paediatric BECs that were differentiated at ALI in the animal-free culture conditions exhibited lower TEER at 4 weeks post-ALI (∼200 Ω·cm^−2^) compared with those previously reported using adult nasal AECs (>300 Ω·cm^−2^) and human airway tissue (trachea and mainstem bronchi, ∼500 Ω·cm^−2^) [11, 32, 33]. Despite this, they achieved some barrier function, reducing paracellular permeability of fluorescently labelled dextran, and showed evidence of specialised cell types, such as ciliated and goblet cells. CBF measurements at 4 weeks post-ALI (7.66±1.95 Hz) were reduced compared with the reported range for nasal cilia (12–16 Hz), but were within range compared with CBF from nasal brush biopsies (4.25–11.63 Hz), human BECs differentiated in PneumaCult™-ALI medium (10.44±0.93 Hz) and brushings from the proximal bronchi of resected lungs (7.1±1.29 Hz) [34–37]. It is worth mentioning that CBF can be affected by environmental factors, for instance, increasing temperatures have been associated with increased CBF in nasal brushings, whereas CBF slows down in ageing ciliated nasal AECs [38–40]. The exact numbers of ciliated and goblet cells in the differentiated epithelium are highly donor dependent. Further experiments using a larger sample size of paediatric BEC donors would provide a more comprehensive understanding of how donor-to-donor variability impacts cellular composition and differentiation capacity in this animal-free model.

Our results demonstrate that RSV propagated in animal-free VP-SFM efficiently infects differentiated BECs and induced a robust inflammatory response, with elevated IL-6 and IL-8 levels in the apical supernatant. Although no direct side-by-side experiments with FBS-containing medium were performed, these results are consistent with previous studies showing that RSV infection induces a strong epithelial pro-inflammatory cytokine response [32, 41–44].

### Conclusion

Animal-free cell culture systems, including serum-free medium and human ECM proteins, offer a promising alternative to traditional animal-derived components. While challenges in differentiation and barrier integrity exist, the ability of these systems to support cell growth and enhance viral propagation highlights their potential for research and therapeutic applications. Importantly, our findings demonstrate that replacing animal-derived components in cell culture systems with animal-free reagents supports effective viral propagation and differentiation of primary airway epithelial cells, highlighting their potential for animal-free reagents in studying RSV pathogenesis and antiviral testing. Further optimisation and validation of these methods will contribute to more ethical, reproducible and human-relevant *in vitro* models.

## Supplementary material

**Please note:** supplementary material is not edited by the Editorial Office, and is uploaded as it has been supplied by the author. Figure S1 00028-2025.SUPPLEMENT Figure S2 00028-2025.SUPPLEMENT2

## References

[1] Weber T, Wiest J, Oredsson S, et al. Case studies exemplifying the transition to animal component-free cell culture. Altern Lab Anim 2022; 50: 330–338. doi:10.1177/02611929221117999 35983799

[2] Wadman M. FDA no longer has to require animal testing for new drugs. Science 2023; 379: 127–128. doi:10.1126/science.adg6276 36634170

[3] Loewa A, Feng JJ, Hedtrich S. Human disease models in drug development. Nat Rev Bioeng 2023: 1, 545–559. 10.1038/s44222-023-00063-3 PMC10173243 37359774

[4] Grimm H, Biller-Andorno N, Buch T, et al. Advancing the 3Rs: innovation, implementation, ethics and society. Front Vet Sci 2023; 10: 1185706. doi:10.3389/fvets.2023.1185706 37396988 PMC10310538

[5] Hoang DM, Pham PT, Bach TQ, et al. Stem cell-based therapy for human diseases. Signal Transduct Target Ther 2022; 7: 272. doi:10.1038/s41392-022-01134-4 35933430 PMC9357075

[6] Canovas D, Bird N. Human AB serum as an alternative to fetal bovine serum for endothelial and cancer cell culture. ALTEX 2012; 29: 426–428. doi:10.14573/altex.2012.4.426 23138512

[7] Shi T, McAllister DA, O'Brien KL, et al. Global, regional, and national disease burden estimates of acute lower respiratory infections due to respiratory syncytial virus in young children in 2015: a systematic review and modelling study. Lancet 2017; 390: 946–958. doi:10.1016/S0140-6736(17)30938-8 28689664 PMC5592248

[8] Renn M, Bartok E, Zillinger T, et al. Animal models of SARS-CoV-2 and COVID-19 for the development of prophylactic and therapeutic interventions. Pharmacol Ther 2021; 228: 107931. doi:10.1016/j.pharmthera.2021.107931 34171328 PMC8219947

[9] Taylor G. Animal models of respiratory syncytial virus infection. Vaccine 2017; 35: 469–480. doi:10.1016/j.vaccine.2016.11.054 27908639 PMC5244256

[10] Deng Y, Herbert JA, Smith CM, et al. An in vitro transepithelial migration assay to evaluate the role of neutrophils in Respiratory Syncytial Virus (RSV) induced epithelial damage. Sci Rep 2018; 8: 6777. doi:10.1038/s41598-018-25167-4 29712964 PMC5928117

[11] Herbert JA, Deng Y, Hardelid P, et al. beta2-integrin LFA1 mediates airway damage following neutrophil transepithelial migration during respiratory syncytial virus infection. Eur Respir J 2020; 56: 1902216. doi:10.1183/13993003.02216-2019 32217648 PMC7406857

[12] Kelsen SG, Mardini IA, Zhou S, et al. A technique to harvest viable tracheobronchial epithelial cells from living human donors. Am J Respir Cell Mol Biol 1992; 7: 66–72. doi:10.1165/ajrcmb/7.1.66 1320903

[13] Maestre-Batlle D, Pena OM, Hirota JA, et al. Novel flow cytometry approach to identify bronchial epithelial cells from healthy human airways. Sci Rep 2017; 7: 42214. doi:10.1038/srep42214 28165060 PMC5292697

[14] Butler CR, Hynds RE, Gowers KH, et al. Rapid expansion of human epithelial stem cells suitable for airway tissue engineering. Am J Respir Crit Care Med 2016; 194: 156–168. doi:10.1164/rccm.201507-1414OC 26840431 PMC5003214

[15] Schindelin J, Arganda-Carreras I, Frise E, et al. Fiji: an open-source platform for biological-image analysis. Nat Methods 2012; 9: 676–682. doi:10.1038/nmeth.2019 22743772 PMC3855844

[16] Grant O, Larken I, Reitemeier S, et al. ciliR: a new R package for determining ciliary beat frequency using fast-Fourier transformation. bioRxiv 2023; preprint [10.1101/2023.12.20.572306].

[17] Bonser LR, Koh KD, Johansson K, et al. Flow-cytometric analysis and purification of airway epithelial-cell subsets. Am J Respir Cell Mol Biol 2021; 64: 308–317. doi:10.1165/rcmb.2020-0149MA 33196316 PMC7909335

[18] Menco BP. Lectins bind differentially to cilia and microvilli of major and minor cell populations in olfactory and nasal respiratory epithelia. Microsc Res Tech 1992; 23: 181–199. doi:10.1002/jemt.1070230208 1421555

[19] Nakamura R, Katsuno T, Kishimoto Y, et al. A novel method for live imaging of human airway cilia using wheat germ agglutinin. Sci Rep 2020; 10: 14417. doi:10.1038/s41598-020-71049-z 32879324 PMC7468155

[20] Fix J, Galloux M, Blondot ML, et al. The insertion of fluorescent proteins in a variable region of respiratory syncytial virus L polymerase results in fluorescent and functional enzymes but with reduced activities. Open Virol J 2011; 5: 103–108. doi:10.2174/1874357901105010103 21966341 PMC3178903

[21] Dewhurst-Maridor G, Simonet V, Bornand JE, et al. Development of a quantitative TaqMan RT-PCR for respiratory syncytial virus. J Virol Methods 2004; 120: 41–49. doi:10.1016/j.jviromet.2004.03.017 15234808

[22] Castagne N, Barbier A, Bernard J, et al. Biochemical characterization of the respiratory syncytial virus p-p and p-N protein complexes and localization of the p protein oligomerization domain. J Gen Virol 2004; 85: 1643–1653. doi:10.1099/vir.0.79830-0 15166449

[23] Hynds RE, Butler CR, Janes SM, et al. Expansion of human airway basal stem cells and their differentiation as 3D tracheospheres. Methods Mol Biol 2019; 1576: 43–53. doi:10.1007/7651_2016_5 27539459

[24] Kuek LE, Lee RJ. First contact: the role of respiratory cilia in host-pathogen interactions in the airways. Am J Physiol Lung Cell Mol Physiol 2020; 319: L603–LL19. doi:10.1152/ajplung.00283.2020 32783615 PMC7516383

[25] Smith CM, Kulkarni H, Radhakrishnan P, et al. Ciliary dyskinesia is an early feature of respiratory syncytial virus infection. Eur Respir J 2014; 43: 485–496. doi:10.1183/09031936.00205312 23520320

[26] Garcia S R, Deprez M, Lebrigand K, et al. Novel dynamics of human mucociliary differentiation revealed by single-cell RNA sequencing of nasal epithelial cultures. Development 2019; 146: dev177428. doi:10.1242/dev.174318 31558434 PMC6826037

[27] Villenave R, Thavagnanam S, Sarlang S, et al. In vitro modeling of respiratory syncytial virus infection of pediatric bronchial epithelium, the primary target of infection in vivo. Proc Natl Acad Sci U S A 2012; 109: 5040–5045. doi:10.1073/pnas.1110203109 22411804 PMC3323997

[28] Ahmed Z, Powell LC, Matin N, et al. Jellyfish collagen: a biocompatible collagen source for 3D scaffold fabrication and enhanced chondrogenicity. Mar Drugs 2021; 19: 405. doi:10.3390/md19080405 34436244 PMC8400217

[29] Miyazaki T, Futaki S, Suemori H, et al. Laminin E8 fragments support efficient adhesion and expansion of dissociated human pluripotent stem cells. Nat Commun 2012; 3: 1236. doi:10.1038/ncomms2231 23212365 PMC3535336

[30] Horani A, Nath A, Wasserman MG, et al. Rho-associated protein kinase inhibition enhances airway epithelial basal-cell proliferation and lentivirus transduction. Am J Respir Cell Mol Biol 2013; 49: 341–347. doi:10.1165/rcmb.2013-0046TE 23713995 PMC3824057

[31] Witkowski TA, Li B, Andersen JG, et al. Y-27632 acts beyond ROCK inhibition to maintain epidermal stem-like cells in culture. J Cell Sci 2023; 136: jcs260990. doi:0.1242/jcs.260990 37698512 10.1242/jcs.260990 PMC10508688

[32] Robinson E, Herbert JA, Palor M, et al. Trans-epithelial migration is essential for neutrophil activation during RSV infection. J Leukoc Biol 2023; 113: 354–364. doi:10.1093/jleuko/qiad011 36807711 PMC11334017

[33] Yonker LM, Mou H, Chu KK, et al. Development of a primary human co-culture model of inflamed airway mucosa. Sci Rep 2017; 7: 8182. doi:10.1038/s41598-017-08567-w 28811631 PMC5557980

[34] Clary-Meinesz C, Mouroux J, Huitorel P, et al. Ciliary beat frequency in human bronchi and bronchioles. Chest 1997; 111: 692–697. doi:10.1378/chest.111.3.692 9118710

[35] Leung C, Wadsworth SJ, Yang SJ, et al. Structural and functional variations in human bronchial epithelial cells cultured in air-liquid interface using different growth media. Am J Physiol Lung Cell Mol Physiol 2020; 318: L1063–L1073. doi:10.1152/ajplung.00190.2019 32208929

[36] Raidt J, Wallmeier J, Hjeij R, et al. Ciliary beat pattern and frequency in genetic variants of primary ciliary dyskinesia. Eur Respir J 2014; 44: 1579–1588. doi:10.1183/09031936.00052014 25186273

[37] Yaghi A, Dolovich MB. Airway epithelial cell cilia and obstructive lung disease. Cells 2016; 5: 40. doi:10.3390/cells5040040 27845721 PMC5187524

[38] Smith CM, Hirst RA, Bankart MJ, et al. Cooling of cilia allows functional analysis of the beat pattern for diagnostic testing. Chest 2011; 140: 186–190. doi:10.1378/chest.10-1920 21193531

[39] Ho JC, Chan KN, Hu WH, et al. The effect of aging on nasal mucociliary clearance, beat frequency, and ultrastructure of respiratory cilia. Am J Respir Crit Care Med 2001; 163: 983–988. doi:10.1164/ajrccm.163.4.9909121 11282777

[40] Nikolaizik W, Hahn J, Bauck M, et al. Comparison of ciliary beat frequencies at different temperatures in young adults. ERJ Open Res 2020; 6: 00477-2020. doi:10.1183/23120541.00477-2020 33263055 PMC7682707

[41] Diaz PV, Gaggero AA, Pinto RA, et al. Levels of inflammatory cytokines and plasma cortisol in respiratory syncytial virus bronchiolitis. Rev Med Chil 2013; 141: 574–581. doi:10.4067/S0034-98872013000500004 24089271

[42] Tabarani CM, Bonville CA, Suryadevara M, et al. Novel inflammatory markers, clinical risk factors and virus type associated with severe respiratory syncytial virus infection. Pediatr Infect Dis J 2013; 32: e437–e442. doi:10.1097/INF.0b013e3182a14407 23804121 PMC3883981

[43] Villenave R, O'Donoghue D, Thavagnanam S, et al. Differential cytopathogenesis of respiratory syncytial virus prototypic and clinical isolates in primary pediatric bronchial epithelial cells. Virol J 2011; 8: 43. doi:10.1186/1743-422X-8-43 21272337 PMC3039598

[44] McNamara PS, Flanagan BF, Hart CA, et al. Production of chemokines in the lungs of infants with severe respiratory syncytial virus bronchiolitis. J Infect Dis 2005; 191: 1225–1232. doi:10.1086/428855 15776367

