# Supplementary material for: Ciliated epithelial cell differentiation at air–liquid interface and respiratory syncytial virus infection using animal-free media and substrates
Source: ERJ Open Res. 2025 Dec 8;11(6):00028-2025. doi: 10.1183/23120541.00028-2025 (PMC12683564; doi:10.1183/23120541.00028-2025)
Supplement: Supplementary file 1 [file 00028-2025.SUPPLEMENT.pdf]

Supplementary Figure 1

a.

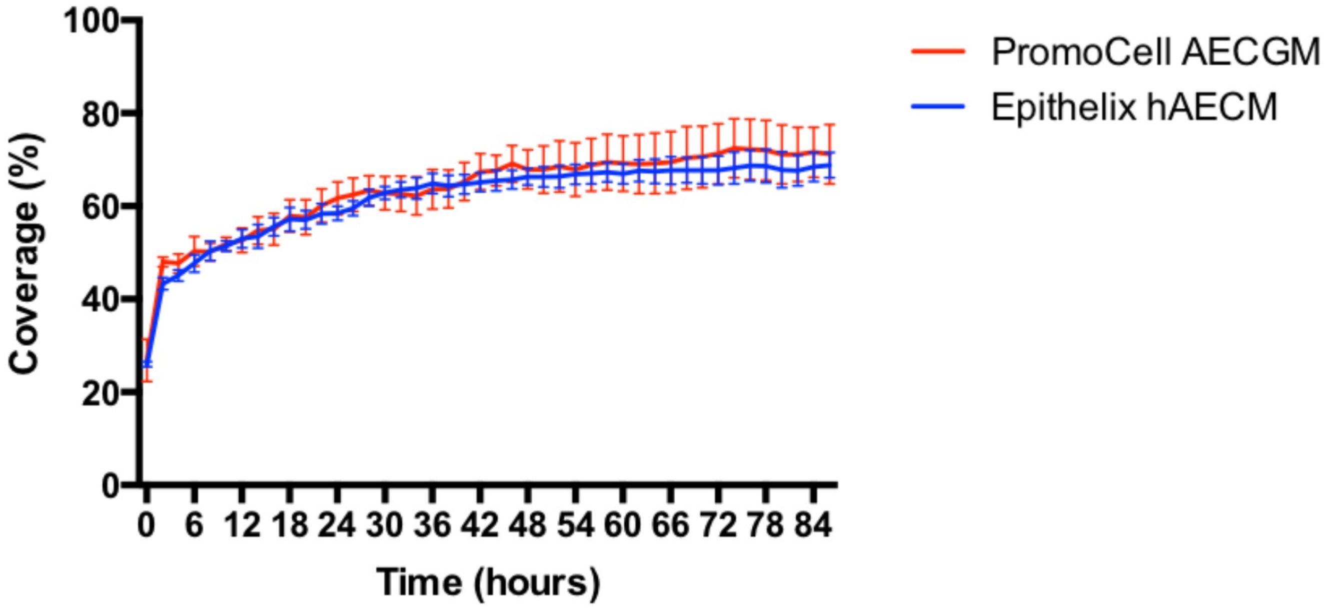

b.

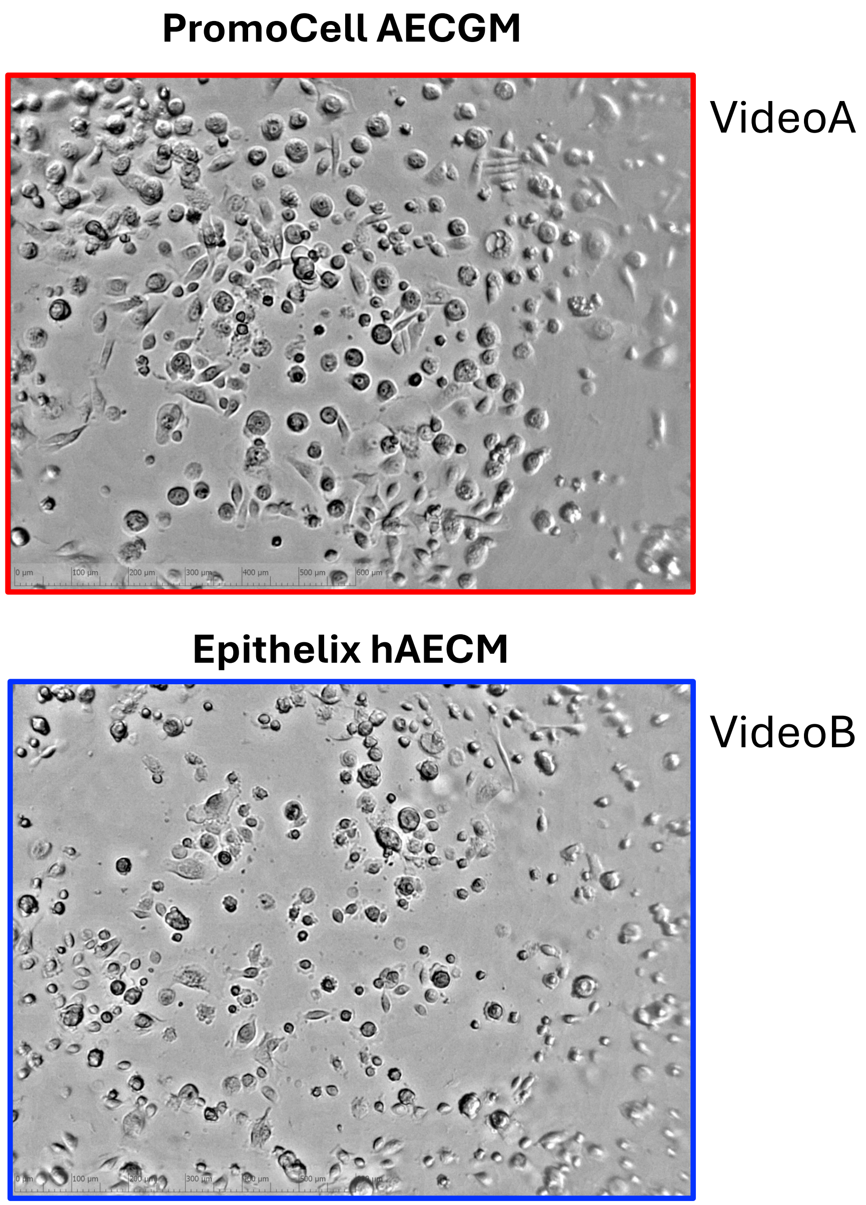

Supplementary Figure 1. Surface area coverage and proliferation of BECs cultured in two different AEC growth medium over time (using the zenCELL owl incubator microscope (LabLogic, UK)). **A** Error bars represent mean ± SEM of *N* = 3 independent experiments. **B** Representative timelapse videos 0-84 h of one region of interest.
