# Supplementary material for: Ciliated epithelial cell differentiation at air–liquid interface and respiratory syncytial virus infection using animal-free media and substrates
Source: ERJ Open Res. 2025 Dec 8;11(6):00028-2025. doi: 10.1183/23120541.00028-2025 (PMC12683564; doi:10.1183/23120541.00028-2025)
Supplement: Supplementary file 2 [file 00028-2025.SUPPLEMENT2.pdf]

Supplementary Figure 2

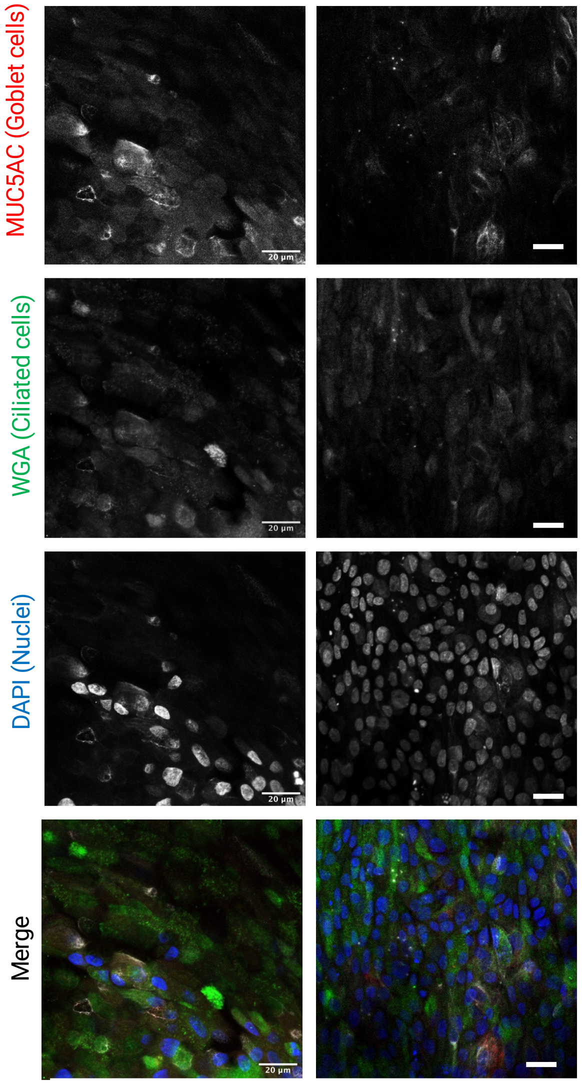

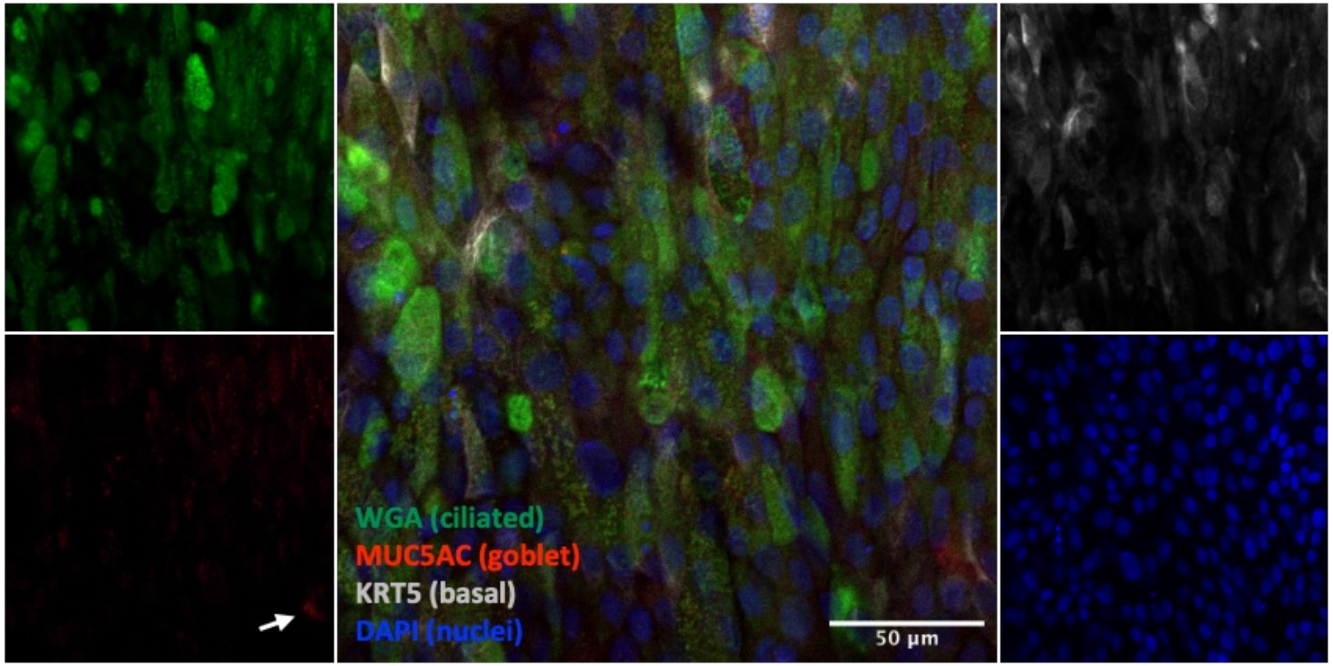

Supplementary Figure 2. Representative IF image to show expression of WGA (green, ciliated cell), MUC5AC (red, goblet cell) and KRT5 (grey, basal cell) staining. Nuclei (blue) counterstained with DAPI stained using animal-free reagents. Scale bars = 25µm (left) or 50µm (above).
